# A novel lower extremity non-contact injury risk prediction model based on multimodal fusion and interpretable machine learning

**DOI:** 10.3389/fphys.2022.937546

**Published:** 2022-09-15

**Authors:** Yuanqi Huang, Shengqi Huang, Yukun Wang, Yurong Li, Yuheng Gui, Caihua Huang

**Affiliations:** ^1^ Research and Communication Center for Exercise and Health, Xiamen University of Technology, Xiamen, China; ^2^ School of Physical Education and Sport Science, Fujian Normal University, Fuzhou, China; ^3^ College of Electrical Engineering and Automation, Fuzhou University, Fuzhou, China; ^4^ Fujian Provincial Basketball and Volleyball Centre, Fuzhou, China

**Keywords:** injury prevention, machine learning, multimodal fusion, injury risk pattern, injury risk prediction

## Abstract

The application of machine learning algorithms in studying injury assessment methods based on data analysis has recently provided a new research insight for sports injury prevention. However, the data used in these studies are primarily multi-source and multimodal (i.e., longitudinal repeated-measures data and cross-sectional data), resulting in the models not fully utilising the information in the data to reveal specific injury risk patterns. Therefore, this study proposed an injury risk prediction model based on a multi-modal strategy and machine learning algorithms to handle multi-source data better and predict injury risk. This study retrospectively analysed the routine monitoring data of sixteen young female basketball players. These data included training load, perceived well-being status, physiological response, physical performance and lower extremity non-contact injury registration. This study partitions the original dataset based on the frequency of data collection. Extreme gradient boosting (XGBoost) was used to construct unimodal submodels to obtain decision scores for each category of indicators. Ultimately, the decision scores from each submodel were fused using the random forest (RF) to generate a lower extremity non-contact injury risk prediction model at the decision-level. The 10-fold cross-validation results showed that the fusion model was effective in classifying non-injured (mean Precision: 0.9932, mean Recall: 0.9976, mean F2-score: 0.9967), minimal lower extremity non-contact injuries risk (mean Precision: 0.9317, mean Recall: 0.9167, mean F2-score: 0.9171), and mild lower extremity non-contact injuries risk (mean Precision: 0.9000, mean Recall: 0.9000, mean F2-score: 0.9000). The model performed significantly more optimal than the submodel. Comparing the fusion model proposed with a traditional data integration scheme, the average Precision and Recall improved by 8.2 and 20.3%, respectively. The decision curves analysis showed that the proposed fusion model provided a higher net benefit to athletes with potential lower extremity non-contact injury risk. The validity, feasibility and practicality of the proposed model have been confirmed. In addition, the shapley additive explanation (SHAP) and network visualisation revealed differences in lower extremity non-contact injury risk patterns across severity levels. The model proposed in this study provided a fresh perspective on injury prevention in future research.

## Introduction

Sports injury is a hot issue in the sports science and sports medicine communities and is also a practical problem that urgently needs to be solved (López-Valenciano et al., 2019). It has previously been observed that sports injuries often occur in team ball games. The injury rate of basketball events has increased annually, especially since the risk of sports injuries in youth groups was extremely high. Non-contact injuries accounted for 47.0% of training injuries in basketball players, the incidence of non-contact injuries among centre players is as high as 86.1%, and 28.0% of non-contact injuries resulted in absences from the training of more than 7 days (Meeuwisse et al., 2003; Agel et al., 2007). Several theories on sports injury prevention have been proposed in sports science and sports medicine communities. Nevertheless, there have been few detailed investigations of injury risk assessment methods based on data analysis that can effectively predict and assess the injury risk of athletes, which significantly limits the development of the athletic ability of elite athletes and the scientific process of sports training. By studying injury risk assessment methods based on data analysis, the risk patterns of sports injuries can be effectively identified and recognised, which is vital for developing good training programs and targeted interventions and reducing sports injury rates.

The practical implementation of injury risk assessment methods based on data analysis requires the establishment of effective injury risk prediction models (Georgios et al., 2019). However, a review conducted by (Ruddy et al., 2019) has pointed out that the data collected during sports training monitoring mainly originated from real-world research environments, which included a large amount of data, many variables, and an uneven distribution of injury samples. This made statistical modelling methods based on parameterization slightly inadequate in the application of injury risk factor discussion and injury risk prediction, hindering the development of injury risk assessment methods based on data analysis, resulting in sports injury prevention strategies that are still based on empirical judgement rather than data (Luo et al., 2020; Fiscutean, 2021). In response to these issues (Fiscutean, 2021), pointed out in the New Viewpoint of Sports Science in Nature that modelling and analysing the relationship between athletes’ training data and sports injury risk using machine learning algorithms would help to assist in predicting athletes’ injury risk and provide a decision basis for athletes’ training load adjustments. This has been the main direction to solve the early warning of sports injury risk. Recently, researchers in sports science and sports medicine communities have shown an increased interest in applying machine learning algorithms to model the injury risk of athletes from different research dimensions (Claudino et al., 2019; Rossi et al., 2022a; Rossi et al., 2022b). For example (Talukder et al., 2016), proposed a sports injury risk prediction model based on the time sliding window and random forest (RF), which could effectively use athletes’ technical and tactical statistics to predict athletes’ injuries during the season. The study noted that average speed, number of games, number of games played, average distance, average game time, and average number of shots may be important variables in predicting injury risk (Rossi et al., 2018). constructed an injury prediction model based on GPS monitoring data of Italian male professional soccer players and decision tree algorithms and successfully predicted approximately 80% of non-contact injuries by the model (Rommers et al., 2020). used the extreme gradient boosting (XGBoost) to predict and model the relationship among pregame athletic quality assessment tests, anthropometric data and injuries in 734 U10 to U15 soccer players and constructed injury risk prediction models that could detect 85% of injury conditions with 85% precision. Extensive research has confirmed that machine learning algorithms can effectively predict sports injuries. However, researchers have not treated this novel method in much detail. First, the granularity of the data still needs further refinement. Most of the existing studies consider the occurrence of injury as the dependent variable without considering the specific injury sites or injury severity (López-valenciano et al., 2017; Rossi et al., 2018; Bryan et al., 2020). Second, there is a lack of injury risk prediction model construction methods for multi-source and multi-modal data. The data types involved in the above studies are mostly longitudinal repeated measures data with multiple time points in a single dimension, or cross-sectional data with a single time point in multiple dimensions. However, with the development of science and technology, data in training practice are characterised by multi-source and multimodal (i.e. containing both longitudinal repeated-measures data and cross-sectional data). It makes the traditional injury risk modelling methods may suffer from insufficient data processing capability when handling data (Baltrvaaitis et al., 2019; Luo et al., 2020). Last, injury risk patterns have not been explored. Sports injuries are the result of a combination of multiple factors. However, due to the limitations of conventional statistical methods and modelling strategies, previous studies have only been able to obtain only information reflecting some factors in the injury risk pattern but not the complete picture of the injury risk pattern (Waterkamp et al., 2016; Bello et al., 2020; Isern-Kebschull et al., 2020). Therefore, this study proposes an injury risk multimodal fusion model with generality, interpretability and ease of implementation based on a multimodal fusion strategy to suit the multi-source, multimodal data processing and analysis needs in training monitoring. This will help coaches and team doctors understand the risk patterns of lower extremity non-contact injuries in basketball teams and are also essential for developing reasonable training plans, adopting targeted interventions, and reducing sports injury rates.

This study further investigates the injury risk prediction method based on data analysis by using routine monitoring data of young female basketball players in Fujian Province. The monitoring indices were divided into multiple modalities based on the evaluation dimensions, and the XGBoost was used to construct unimodal submodels. The RF was used to fuse the decision results of submodels of different modalities and propose the final injury risk prediction model. The validity of the proposed model was determined by comparing it with a unimodal submodel and a prediction model using a traditional fusion approach. SHAP was also used to analyse the weights of monitoring indices in the submodels and the weights of submodels in the fusion model to explain injury risk patterns.

## Materials and methods

Sixteen young female basketball players (age: 16.6 ± 1.3 years, height: 175.4 ± 6.3 cm, weight: 65.7 ± 6.2 kg, years of training: 3.3 ± 1.7 years) participated in the study. All players were affiliated with the Fujian Provincial Basketball and Volleyball Centre. The data in this study came from 20 weeks of routine monitoring of the players (November 2020 to April 2021), including training load, perceived well-being status, physiological responses, physical performance and player injuries. The study was conducted with the approval of the Fujian Provincial Basketball and Volleyball Centre. All participants provided fully informed consent to participate in this study by signing a written consent form.

### Data collection

Monitoring and calculation of internal training load. This study used the Borg-10 ratings of perceived exertion (RPE) scale designed by (Foster et al., 1995) to quantify the perceived exertion level of players after each training session. The validity and reliability of this quantification method have been confirmed in numerous studies (Chen et al., 2002). Within 30 min after each training session or competition, the player was verbally asked how tired they were after completing that session. The duration between the start of each training session and the end of the training was recorded. Eq. 1 was used to calculate the session rating of perceived exertion (sRPE) of a single training session to quantify the training load of each training session. The training duration unit was minutes, and the RPE was an arbitrary unit (AU). In the study, the daily training load of each player was calculated based on the quantified data of the load of each training session, taking the training day as the unit.

sRPE=duration×RPE (1)

Monitoring and calculation of perceived well-being status. The perceived well-being status questionnaire designed by (Hooper et al., 1995) was used to quantify players’ perceived well-being status in the morning on training days. The scale used a Likert 5-level score, and the scoring items included fatigue, sleep quality, muscle soreness, stress level, and training desire. Each item ranged from “very bad” to “very good”, with a value of 1–5 points. The daily menstrual conditions of players were inquired about and recorded (0 is negative/no period; 1 is positive/period).

Physical performance. Refer to the physical performance test and evaluation plan in “Sports Injury Management” (Joyce and Lewindon, 2016). In this study, the squat 1RM test was selected to assess the player’s maximum lower extremity muscle strength; the 15 m × 17 round shuttle run test was selected to assess the player’s speed endurance; the 5.8 m × 6 round shuttle run test was selected to assess the player’s agility, and the maximum vertical jump test was selected to assess the player’s explosive and jumping ability.

Physiological response. Urine was collected from players every Wednesday after training. Protein, specific gravity, blood, urobilinogen, pH, and ketones in urine were detected using the Siemens Clinitek Status Urine Analyser to assess the physiological state of players. The assignment of the urine test results is shown in Table 1.

**TABLE 1:** Assignment of indices and units.

Index	Assignment	Frequency	Unit
sRPE	Original value input	day	AU
Menses	No = 0, Yes = 1	day	AU
Fatigue	Original value input	day	AU
Sleep Quality	Original value input	day	AU
Muscle Soreness	Original value input	day	AU
Stress Levels	Original value input	day	AU
Desire	Original value input	day	AU
Urine Protein	Negative = 1; Microscale = 2; 0.3 g/L = 3; 1 g/L = 4; 3 g/L = 5	1-week	AU
Urobilinogen	3.2 mg/dl = 1; 16 mg/dl = 5; 33 mg/dl = 10	1-week	AU
Urine pH	Original value input	1-week	AU
Urine Specific Gravity	≤1.025 = 1; ≥1.030 = 2	1-week	AU
Urine Blood	Negative = 1; Microscale = 2; Ca25 Ery/µL = 3; Ca80 Ery/µL = 4; Ca200 Ery/µL = 6	1-week	AU
Urine Ketones	Negative = 1; Microscale = 2; 1.5 nmol/L = 3	1-week	AU
Squat 1RM	Original value input	4-weeks	kg
15 m × 17 Shuttle Run	Original value input	4-weeks	s
5.8 m × 6 Shuttle Run	Original value input	4-weeks	s
Maximum Vertical Jump	Original value input	4-weeks	cm
Injury Severity	Negative = 0; 0–3 = 1; 4–7 = 2; 8–28 = 3; ≥29 = 4	day	AU

Injury registration. Referring to the injury data collection procedure of (Fuller et al., 2006), injuries were diagnosed by medical personnel from the Fujian Provincial Basketball and Volleyball Centre through medical examination and other methods. The injury registry recorded information such as location, nature, type, and occurrence of injury (contact, non-contact) and diagnosis mode. Referring to the definition in the literature (Bahr et al., 2020), this study defined lower extremity non-contact injuries (LENCIs) as injuries to the lower extremity area caused by mechanisms other than direct contact, including overuse injuries and chronic injuries. The lower extremity included the hips, thighs, knees, calves, ankles and feet. Referring to the definition by (Enright et al., 2019), the severity of the injury was classified according to the time missed from training as minimal (0–3 days), mild (4–7 days), moderate (8–28 days) and severe (≥29 days) and assigned a value of 1–4, respectively.

### Data Processing

**FIGURE 1:**
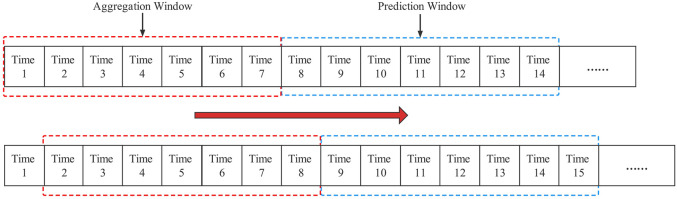
Schematic diagram of the aggregation and prediction sliding windows.

Time sliding window. Research reports showed that the players’ stimulus-response to training load and perceived well-being recovery had the characteristics of accumulation and decay over time. At the same time, there may be a delay between peak training load fluctuations and increased risk of injury (Hulin et al., 2016; Schwellnus et al., 2016; Soligard et al., 2016; Watson et al., 2016). Therefore, this study used the time sliding window technique to create an aggregation sliding window and a prediction sliding window to preprocess the dataset (Figure 1) and perform statistical calculations on the variables within the aggregation sliding window (Talukder et al., 2016). The training monotony (TM) calculation method proposed by (Foster et al., 1995) was referenced to calculate the degree of training load change in the aggregation sliding window (Eq. 2).

$$\mathrm{TM}=\frac{\frac{1}{7}\left(\sum_{i=1}^{7}\mathrm{Loa}d_{i}\right)}{\sqrt{\frac{1}{7}\sum_{i=1}^{7}{\left(\mathrm{loa}d_{i}-\frac{1}{7}\left(\sum_{i=1}^{7}\mathrm{Loa}d_{i}\right)\right)}^{2}}}$$ (2)

The average of the training load index and the perceived well-being index was calculated using the exponentially weighted moving average (EWMA) suggested by (Bourdon et al., 2017) (Eq. 3).

$$\mathrm{EWM}A_{\mathrm{today}}=\frac{2}{N+1}\times \mathrm{Loa}d_{\mathrm{today}}+\left(1-\frac{2}{N+1}\right)\times \mathrm{EWM}A_{\mathrm{yesterday}}$$ (3)

Notably, since the player’s perceived well-being status questionnaire used in the study used a 5-point Likert scale, the score value was low, and the daily variation range was small. Hence, the indices of the change trends of players’ perceived well-being were not calculated. Additionally, since there was no uniform standard for the selection of the time sliding window length, referring to the existing research reports, the aggregation sliding window time parameter was set to 7 days, and the prediction sliding window time parameter were set to 7 days (Hulin et al., 2013; Hulin et al., 2016; Malone et al., 2018).

Dataset division. The data collection in training practice was easily affected by various factors, such as coach cooperation, player compliance and research sustainability. In addition, the data collection frequencies of the training load quantification, perceived well-being status, physiological response and physical performance test in the original dataset were different. The physiological response and physical performance data were missing in the complete time series (Table 1). If these indices were to be removed, this could result in missing information on physiological adaptations and exercise capacity. This study reconstructed the original dataset to generate dataset A with training weeks as the collection frequency and dataset B with training days as the collection frequency. Among them, the missing values in the physiological response data and physical performance test data of dataset B were filled by the adjacent value imputation method at the individual level.

Z-score normalization. Since each player is an independent individual, there are significant differences in the stimulation response to the training load and the perceived well-being recovery of different players. Therefore, This study normalizes the independent variables using the Z-score transform (Eq. 4) for each athlete to facilitate cross-sectional comparisons.

$$Z=\frac{X-\mu }{\sigma }$$ (4)

where x is the original data, *μ* is the mean value of the original data, and *σ* is the standard deviation of the original data.

Class imbalance processing. People with potential risks are the focus of injury risk assessment. However, because the injury that occurred in the actual situation has a largely skewed distribution, there is a class imbalance problem, which causes the model to fail to correctly classify the minority class samples (Han et al., 2005). The synthetic minority oversampling technique (SMOTE) was used in the study to synthetically sample the training set in each fold of the cross-validation. To reduce the negative impact of the class imbalance problem on model training. The SMOTE is an improved scheme based on the random oversampling that can effectively solve the problem of insufficient model generalization caused by the random oversampling (Chawla et al., 2002). The algorithm obtained its k-nearest neighbours by calculating the Euclidean distance from each minority class sample d to all the minority class samples. A sampling ratio was set according to the sample imbalance ratio, several samples $d_{n}$ were selected from the k-nearest neighbours of each minority class sample, and a new sample $d_{new}$ was generated by Eq. 5.

$$d_{\mathrm{new}}=d+\mathrm{rand}\left(0,1\right)*\left(d-d_{n}\right)$$ (5)

### Model construction

**FIGURE 2:**
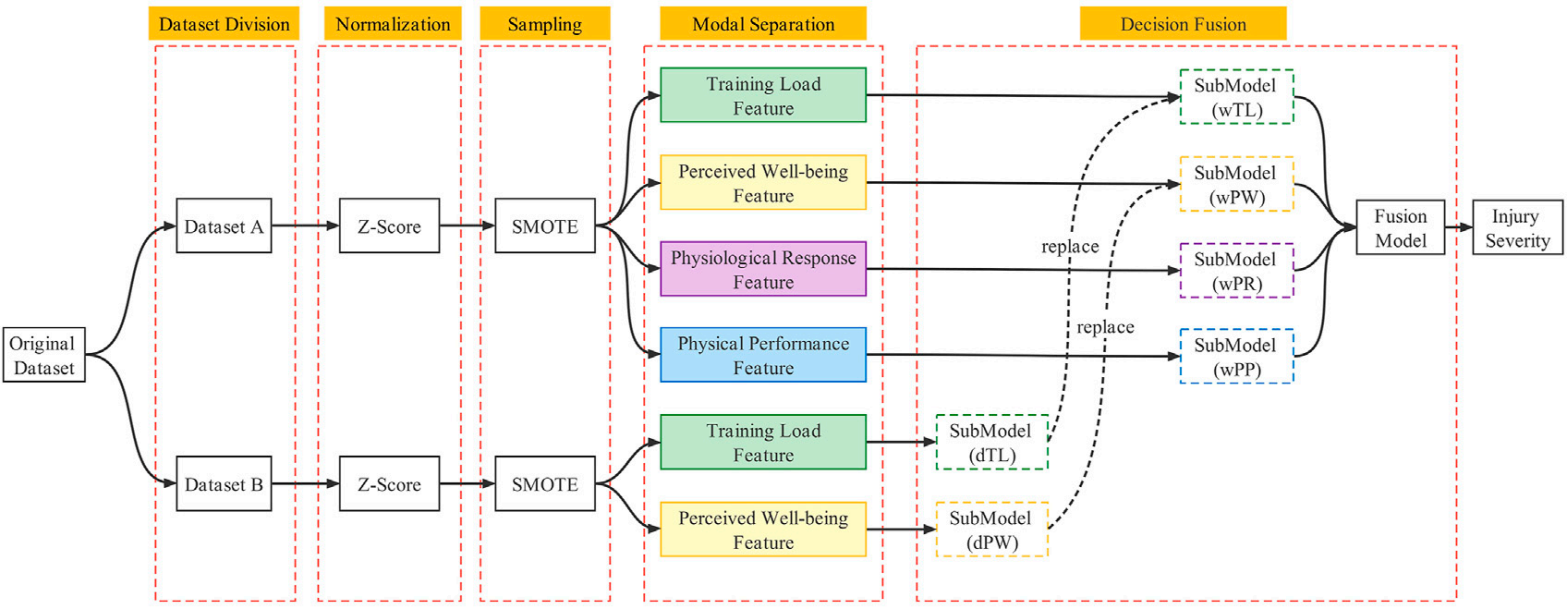
Schematic diagram of the multimodal model architecture.

In this study, the monitoring indices were divided into four modalities based on the evaluation purpose: training load, perceived well-being status, physiological response and physical performance test. Use the occurrence of a LENCI in the next week as the dependent variable. The proposed multimodal fusion model construction process for LENCI risk prediction is shown in Figure 2. First, the submodels of each modality were initially constructed using dataset A. The decision-level fusion of the decision results for each submodel was made to determine the model parameters of the fusion model, which was named wFusionModel. Second, the submodels for training load and perceived well-being states were constructed using dataset B. Finally, it was replaced with the submodels of training load and perceived well-being status in wFusionModel to form the final injury risk prediction model, which was named dFusionModel.

The XGBoost was chosen to construct the unimodal submodel in the study. XGBoost is a machine learning further optimized by (Chen and Guestrin, 2016) based on gradient boosting decision tree (GBDT). The algorithm enhanced the classification ability by integrating the prediction results of multiple decision tree models and making the predicted values of samples as close to the actual values as possible, with better prediction performance and training speed. Its objective optimization function is shown in Eq. 6.

$$\mathrm{Obj}=\overset{N}{\underset{n=1}{\sum }}\left(L\left(y_{n},{\hat{y}}_{n}\right)+\text{Ω}\;\left(f_{n}\right)\right)$$ (6)

$y_{n}$ in Eq. 6 is the actual value and ${\hat{y}}_{n}$ is the predicted value of the model output. The first part of the Equation $L\left(y_{n},{\hat{y}}_{n}\right)$ represents the loss function of the actual and predicted values, which is a differentiable convex loss function that measures the difference between the ${\hat{y}}_{n}$ and $y_{n}$ . The second part $\text{Ω}\;\left(f_{n}\right)$ is a regular term added to control the complexity of the model. The additional regularization term helps to smooth the final learnt weights to avoid over-fitting. The selection of hyperparameters for the models is shown in Supplementary File.

The RF was chosen to construct a multimodal fusion model. The algorithm took m samples of the training set using the bootstrap method with randomized put-back, and random features were selected for each decision tree based on bagging. These m samples were used to build m decision tree models. Eventually, the results were voted upon by these decision tree models (Breiman, 2001). Since randomness was introduced in selecting samples and feature subspaces, the overfitting problem can be better avoided and improve classification accuracy. The decision function of the RF is shown in Eq. 7.

$$H\left(x\right)=\mathrm{argmax}\underset{k}{\sum }I\left[h_{k}\left(x\right)=y\right]$$ (7)

In Eq. 7, $h_{k}\left(x\right)\;$ is the decision tree model,  y is the classification result of the decision tree, and I(·) is the index function. Since there was a class imbalance problem in the dataset, this study improved the impact of the class imbalance problem on model construction by introducing a sample weight parameter in the RF (Chen, 2004), as shown in Eq. 8.

$$\mathrm{weight}=\frac{n\_\mathrm{sample}}{n\_\mathrm{class}\times N}$$ (8)

In Eq. 8, n_sample is the total sample size, n_class is the number of label categories, and N is the number of samples per category.

### Model validation

This study used a 10-fold stratified cross-validation evaluation strategy to evaluate the model’s performance proposed in the study. The original dataset was randomly divided into ten subsets, and the ten subsets were used as the validation set in turn, while the remaining subsets were used as the training set of the model. The average of the model performance evaluation results after ten iterations was calculated as the model’s overall performance. Experiments were conducted in two ways to illustrate the model’s validity in this study.

**FIGURE 3:**
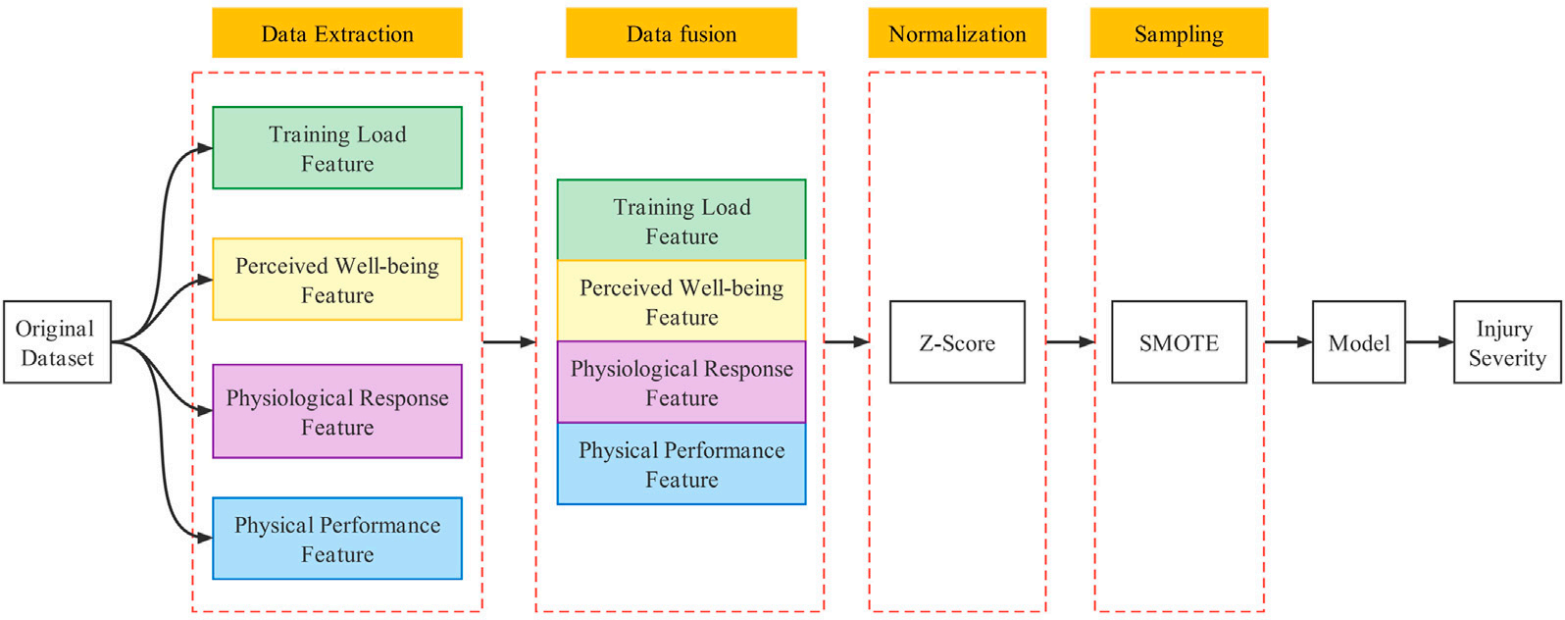
Comparison of reference integration solutions.

1) Comparison with unimodal submodels: We compared the performance of different unimodal submodels and multimodal fusion models with unimodal submodels.
2) Comparison with different fusion schemes: No research has been reported on multimodal fusion strategies in studying sports injury risk prediction models. Therefore, the proposed fusion model in this study was compared with the traditional data integration approach to illustrate the effectiveness of the model. The model building process of the traditional data integration approach is shown in Figure 3. By fusing features of different modalities, they were processed by data normalization and synthetic sampling before being input into the model.

### Model evaluation

In the problem of injury prediction, the cost of missed diagnosis was much higher than injury misdiagnosis and using accuracy alone as a model evaluation index was not appropriate. Therefore, this study used Precision, Recall and F2-score as indices for model performance. They were calculated in the following manner:

$$\mathrm{Precision}=\frac{\mathrm{TP}}{\mathrm{TP}+\mathrm{FP}}$$ (9)

$$\mathrm{Recall}=\frac{\mathrm{TP}}{\mathrm{TP}+\mathrm{FN}}$$ (10)

$$F2-\mathrm{score}=\frac{5\times \mathrm{Precision}\times \mathrm{Recall}}{4\times \mathrm{Precision}+\mathrm{Recall}}$$ (11)

TP, FP, TN, and FN indicate true positives, false positives, true negatives, and false negatives. It is worth noting that the predictor variables in this study are multi categorical variables, and simply calculating the global indices by counting the total number of true positives, false negatives and false positives or using rolling averages to calculate the performance assessment indices for each label is not a useful for evaluating model performance. Thus, this study calculated indices for each label and found their average weighted by support (the number of actual instances for each label) as the final result of the model evaluation.

In addition, the prediction model allowed us to classify exposure situations into two categories: predicted positive and required intervention and predicted negative and did not require intervention. True-positive (TP) exposure and false-positive (FP) exposure were possible within the predicted positive situation. Interventions for the true-positive exposure situation will bring benefits, while interventions for the false-positive exposure situation will cause unnecessary wastage of medical resources and affect the training pace and schedule. Therefore, the $p_{t}$ (probability threshold) of the model needed to be evaluated to assess the clinical utility in training practice. Our research used decision curve analysis (DCA) to evaluate the prediction model (Vickers and Elkin, 2006; Vickers and Holland, 2021). The net benefits of the positive group (Eq. 12) and the negative group (Eq. 13) were calculated to determine the net benefit of the intervention for all predicted positive exposures (Eq. 14) by determining the numerical relationship between the output probability $p_{i}$ and $p_{t}$ of the prediction model. The decision curve was drawn using the probability threshold as the horizontal coordinate and the net benefit as the vertical coordinate.

$$\mathrm{net benefit treated}=\frac{\mathrm{TP}}{n}-\frac{\mathrm{FP}}{n}\times \left(\frac{p_{t}}{1-p_{t}}\right)$$ (12)

$$\mathrm{net benefit untreated}=\frac{\mathrm{TN}}{n}-\frac{\mathrm{FN}}{n}\times \left(\frac{1-p_{t}}{p_{t}}\right)$$ (13)

$$\mathrm{net benefit treat all}=\frac{\mathrm{TP}+\mathrm{FN}}{n}-\frac{\mathrm{TN}+\mathrm{FP}}{n}\times \left(\frac{p_{t}}{1-p_{t}}\right)$$ (14)

### Features importance

Unlike classical statistical modelling methods, XGBoost is a black-box model based on gradient boosting, and its internal working mechanism is challenging to understand. However, the interpretability of the model is very important in training practice. An injury risk model must be understandable and interpretable. Ideally, it should be able to explain the complete logic that provides the corresponding decision to all parties involved. This can help coaches and team doctors develop good training programs and adopt targeted interventions (Ruddy et al., 2019). Therefore, this study used shapley additive explanations (SHAP) for attribution analysis of the prediction model (Lundberg et al., 2020), calculating the absolute weight of each variable according to Eq. 15. We calculate the relative weight of each variable (i.e. the ratio of the absolute weight of a single variable to the sum of the absolute weights of all variables) to facilitate cross-sectional comparisons. We performed model construction, training, validation and analysis of important variables in the Python 3.6 programming environment.

$$\mathrm{Mean}[\mathrm{SHAP}]=\frac{\sum_{i=1}^{N}\left({\left|\mathrm{SHAP}\right|}_{i}\right)}{N}$$ (15)

### Network visualisation

Previous studies have reported that intricate interactions between injury risk factors may allow for differences in the pattern of LENCI risk at different severities levels. In this study, the relationship between the marginal effects of different variables was described in the form of a network to reveal the different levels of LENCI risk pattern. This study assumes that the marginal effects of the variables on injury risk are statistically correlated, and the Spearman correlation coefficient was used as a measure of statistical correlation to analyse the marginal effects of the different variables. The network was plotted using concentric nodes to facilitate cross-sectional comparisons.

### Statistical analysis

Statistical analysis of the data was carried out using STATA 15.0 software. Welch’s *t*-test was used to test for differences in LENCI risk for each index at different injury severities. Differences in model performance and the weights of variables in the models were analysed using Welch’s analysis of variance (ANOVA). All hypothesis tests were conducted using two-sided hypothesis tests, setting *α* in the hypothesis test to 0.05 and considering *p* > 0.1 as not significant, *p* < 0.1 as marginally significant, *p* < 0.05 as significant and *p* < 0.01 as highly significant.

## Results

### Dataset and details of LENCIs

Twenty-seven LENCIs were recorded during the study period, accounting for 62.8% of the total injuries. Among them, approximately 14.8% were non-contact injuries of the knee, 18.5% were non-contact injuries of the thigh, 37.0% were non-contact injuries of the lower leg, and 29.6% were non-contact injuries of the foot. The LENCIs severity is shown in Table 2. Most LENCIs resulted in 1–3 days of missed training, and only 18.5% of LENCIs resulted in more than 4 days of missed training.

**TABLE 2:** Descriptive information on the incidence of non-contact injuries of all lower extremities.

	1–3 days minimal	4–7 days mild	8–28 days moderate	>29 days severe	Count
Hip	0	0	0	0	0 (0.0)
Knee	2	2	0	0	4 (14.8)
Thigh	5	0	0	0	5 (18.5)
Calf	10	0	0	0	10 (37.0)
Ankle	0	0	0	0	0 (0.0)
Foot	5	2	1	0	8 (29.6)

**FIGURE 4:**
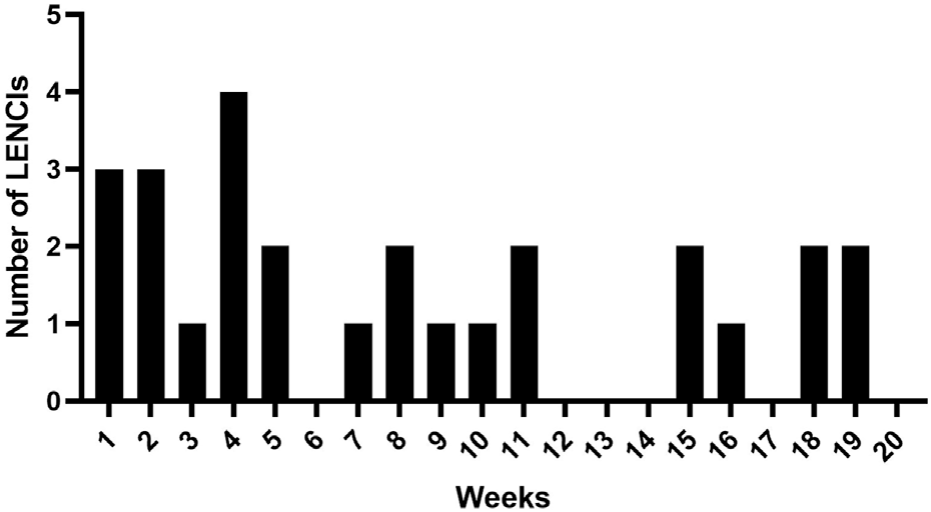
A week of LENCI occurrence.

The number of LENCIs per week is shown in Figure 4. The incidence of LENCIs at weeks 1–5 of routine monitoring was 48.2%. LENCIs in weeks 6–14 and weeks 15–20 accounted for 25.9 and 25.9% of total LENCIs respectively.

The raw data were preprocessed using the time-sliding window algorithm. Due to the calculation needs of the time-sliding window algorithm, the data from the first and last weeks were excluded. At the same time, because urine metabolism during the menstrual period of female athletes will affect the assessment of functional status, urine data during this period were excluded. Descriptive analysis was conducted on the preprocessed dataset, in which the training load and perceived well-being data contained a total of 1813 valid data, the urine data contained 267 valid data, and the physical performance test contained 64 valid data. The basic information of the data of each index is shown in Table 3.

**TABLE 3:** Distribution of each variable in the primary dataset.

Encoding	Feature	Mean ± SD	Minimum	Maximum	N
PW-1	Menses	0.160 ± 0.367	0	1	1813
PW-2	Fatigue (EWMA)	3.019 ± 0.409	2.004	4.570	1813
PW-3	Sleep (EWMA)	3.094 ± 0.448	2.049	4.500	1813
PW-4	MS (EWMA)	3.252 ± 0.453	1.381	4.380	1813
PW-5	Stress (EWMA)	3.044 ± 0.537	1.157	4.410	1813
PW-6	Desire (EWMA)	2.988 ± 0.173	1.610	3.980	1813
TL-1	TM (sRPE)	1.525 ± 0.468	0.267	2.690	1811
TL-2	sRPE (EWMA)	1083.5 ± 265.0	13.885	1685.7	1813
PR-1	Urine Protein	1.719 ± 1.011	1	4	232
PR-2	Urobilinogen	2.056 ± 2.343	1	10	232
PR-3	Urine pH	6.727 ± 0.624	5	8	232
PR-4	Urine Specific Gravity	2.446 ± 0.498	2	3	232
PR-5	Urine Blood	1.854 ± 1.305	1	5	232
PR-6	Urine Ketones	1.330 ± 0.640	1	3	232
PP-1	Squat 1RM	80.592 ± 17.535	60	110	59
PP-2	5.8 m × 6 Shuttle Run	9.747 ± 0.603	8.69	11	59
PP-3	15 m × 17 Shuttle Run	67.637 ± 1.946	63.78	74.16	59
PP-4	MVJ	284.732 ± 6.738	267	295	59

### Model performance evaluation of fusion models

Prediction models were constructed using Table 3 as independent variables and the severity of LENCI in the coming week as dependent variables (including non-injured, minimal LENCI risk and mild LENCI risk). According to the multimodal fusion model construction process proposed in this study, the original dataset was reconstructed to generate dataset A with the training week as the acquisition frequency and dataset B with the training day as the acquisition frequency. The output variable imbalance ratios in datasets A and B were 81:5:1 and 80:5.3:1, respectively. The XGBoost was used to construct submodels for each mode, and the RF was used to fuse the decision results of submodels of different modalities. The performance levels of the submodel and the fusion model are shown in Table 4.

**TABLE 4:** Performance levels of submodels and fusion models in dataset B.

Model	Dimension	Weighted-average precision	Weighted-average recall	Weighted-average F2-score
SubModel (wPW)	Perceived Well-being	0.8657 ± 0.0305	0.8118 ± 0.1141	0.8172 ± 0.1011
SubModel (wTL)	Training Load	0.8776 ± 0.0572	0.7355 ± 0.0702	0.7589 ± 0.0646
SubModel (wPR)	Physiological Response	0.8605 ± 0.0468	0.7315 ± 0.1458	0.7507 ± 0.1306
SubModel (wPP)	Physical Performance	0.8601 ± 0.0325	0.8352 ± 0.0410	0.8399 ± 0.0378
wFusionModel		0.9835 ± 0.0521	0.9731 ± 0.0851	0.9750 ± 0.0792

**FIGURE 5:**
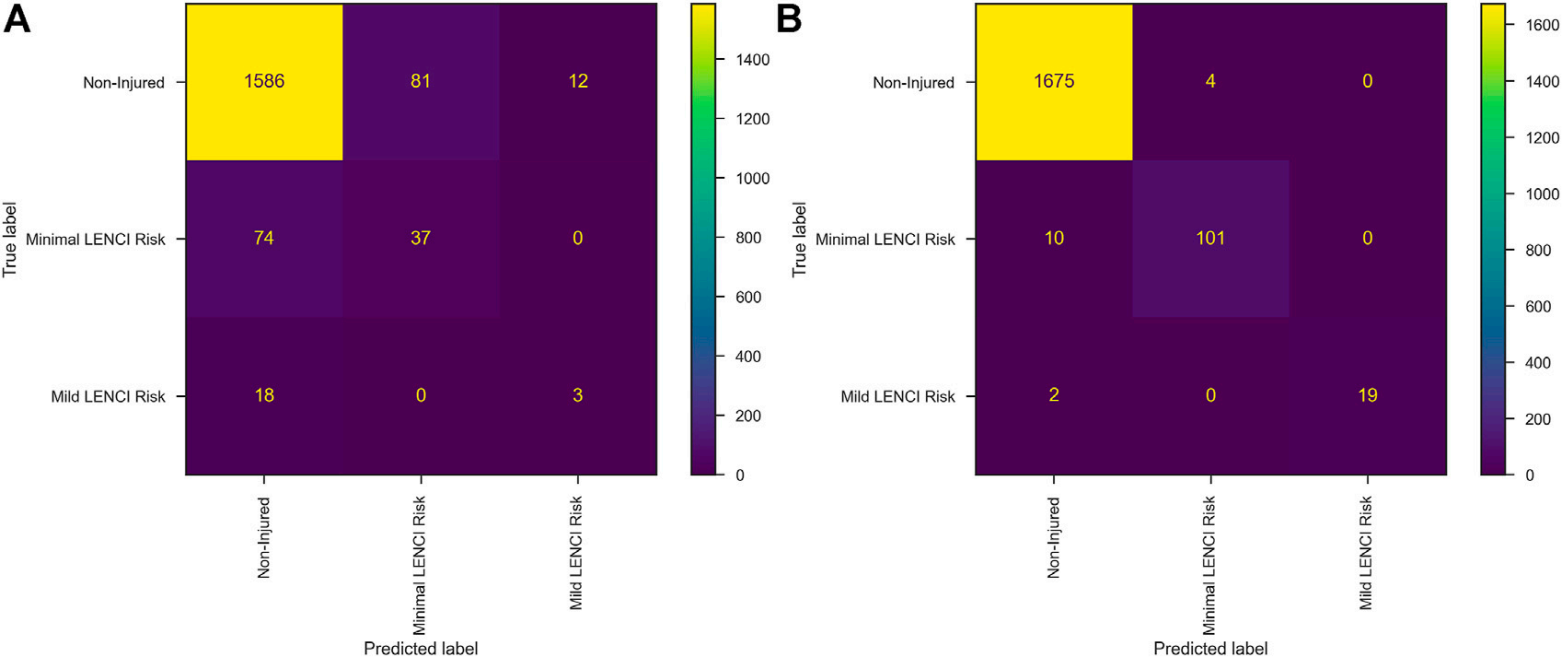
Confusion matrix: **(A)** wFusionModel; **(B)** dFusionModel.

The wFusionModel constructed based on dataset A was used to predict dataset B. The Precision of the model was 0.9012 ± 0.0287, the Recall was 0.8978 ± 0.0507, and the F2-score was 0.8960 ± 0.0464. The confusion matrix is shown in Figure 5A. WFusionModel has many missed and misdiagnosed cases predicting minimal and mild LENCI risk.

The submodel was trained using dataset B’s perceived well-being and training load data. The Precision of the SubModel (dPW) constructed based on the perceived well-being data in dataset B was 0.8754 ± 0.0321, the Recall was 0.8166 ± 0.1354, and the F2-score was 0.8211 ± 0.1188. The Precision of the SubModel (dTL) constructed based on the training load data in dataset B was 0.8663 ± 0.0143, the Recall was 0.8183 ± 0.0345, and the F2-score was 0.8271 ± 0.0301. The results of Welch’s ANOVA showed that the performance levels of SubModel (dPW) and SubModel (dTL) were significantly better than the submodel constructed using the perceived well-being data and training load data from dataset A. SubModel (dPW) and SubModel (dTL) were replaced with SubModel (wPW) and SubModel (wTL) to form the dFusionModel.

**FIGURE 6:**
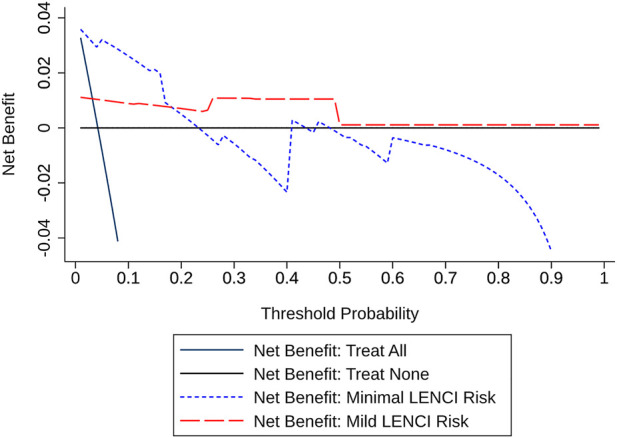
Decision curve analysis.

The dFusionModel’s Precision was 0.9881 ± 0.0423, the Recall was 0.9912 ± 0.0312, and the F2-score was 0.9903 ± 0.0348 by 10-fold cross-validation. dFusionModel’s confusion matrix is shown in Figure 5B. The performance evaluation indices of the dFusionModel model were better than those of the wFusionModel (*p* < 0.01). The decision curve analysis of dFusionModel is shown in Figure 6.

### Comparison between the fusion and integration schemes

This study compared the proposed fusion model with traditional data integration methods. The logistic regression (LR), support vector machine (SVM), k-nearest neighbour (KNN), Gaussian Naïve Bayes (NB), decision tree (DT), RF and XGBoost algorithms are commonly used in research reports, were selected as the base classifiers for the data integration scheme. Furthermore, to compare these classifiers’ ability to identify different levels of LENCI risks, we constructed a dummy classifier (DC) which randomly assigns a class to an example by respecting the distribution of the classes. The experimental results are shown in Table 5. The performance evaluation indices of the dFusionModel proposed in this study were better than those of the prediction models constructed by the data integration scheme. This showed that the fusion model proposed in this study could obtain more accurate results in predicting the severity of LENCI in adolescent female basketball players in Fujian Province. Details of precision, recall and F2 scores for all categories can be found in the Supplementary File.

**TABLE 5:** Performance evaluation results of the fusion and integration schemes in dataset A.

Model	Weighted-average Precision	Weighted-average Recall	Weighted-average F2-score
DC	0.8670 ± 0.0143	0.3506 ± 0.0116	0.3857 ± 0.0089
LR	0.8906 ± 0.0223	0.5638 ± 0.1150	0.5916 ± 0.1048
SVM	0.9206 ± 0.0269	0.9045 ± 0.0351	0.9050 ± 0.0330
KNN	0.8961 ± 0.0175	0.8023 ± 0.0365	0.8140 ± 0.0322
NB	0.9007 ± 0.0260	0.6223 ± 0.1208	0.6422 ± 0.1163
DT	0.9026 ± 0.0260	0.8244 ± 0.1105	0.8324 ± 0.1003
RF	0.9169 ± 0.0355	0.9183 ± 0.0898	0.9152 ± 0.0833
XGBoost	0.9141 ± 0.0322	0.8813 ± 0.0600	0.8835 ± 0.0517
dFusionModel	0.9881 ± 0.0423	0.9912 ± 0.0312	0.9903 ± 0.0348

### Feature importance

**FIGURE 7:**
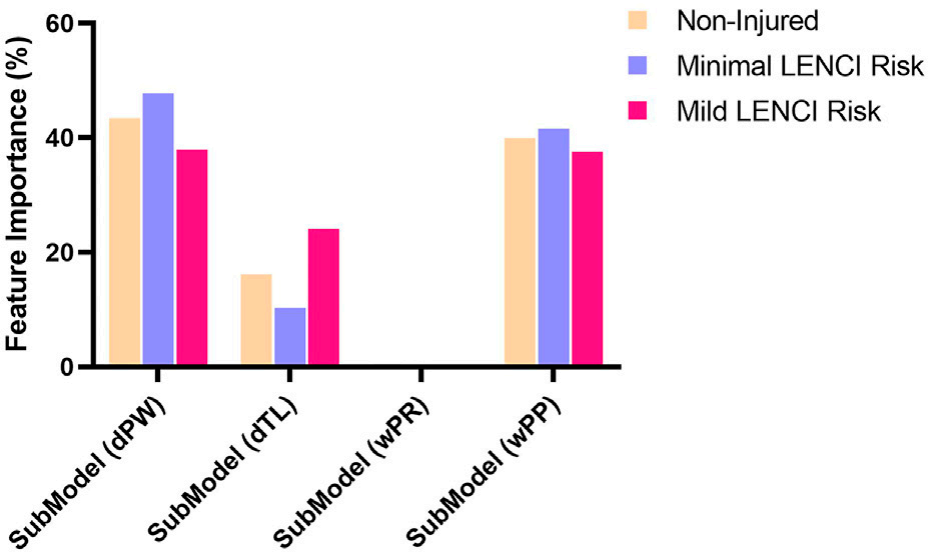
The feature importance of the dFusionModel.

Welch’s ANOVA was used to perform the variation analysis of the relative weights of each submodel in the dFusionModel model. The results showed (Figure 7) that the weights of SubModel (dPW), SubModel (dTL), and SubModel (wP) in different degrees of injury risk were significantly different (*p* < 0.01), while the weights of SubModel (wU) in different degrees of injury risk were not significantly different (*p* > 0.05). SubModel (dPW) and SubModel (wU) had higher weights, indicating that perceived well-being and physical performance are important factors affecting LENCI risk.

**FIGURE 8:**
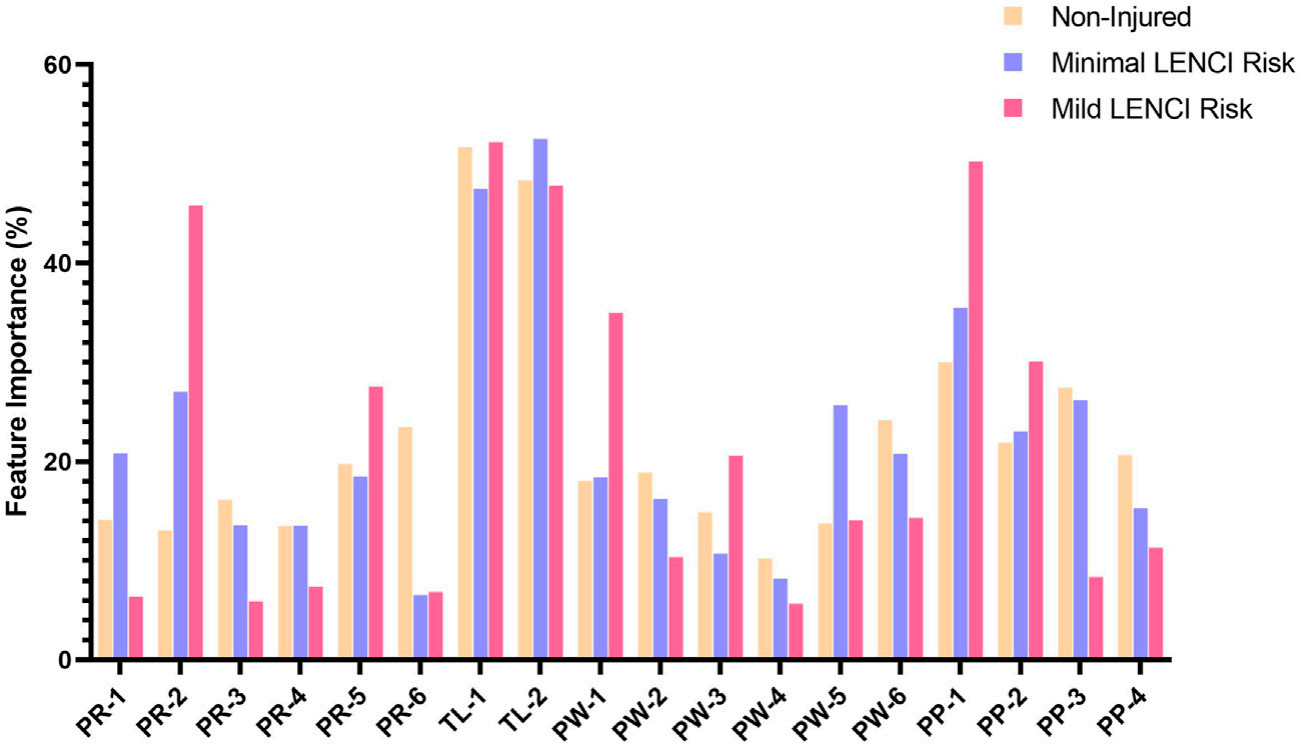
The feature importance of each submodel.

Attribution analysis was performed on the submodels in dFusionModel using SHAP, evaluating the weight of each feature in the submodels. Figure 8 shows the relative weights of each feature in the submodel. Analysis of variance in the relative weights of indices in each classification using Welch’s ANOVA revealed that, compared to the situation in which young female basketball players in Fujian Province did not present a risk of LENCI when presenting a risk of minimal LENCI, the *stress (EWMA)* (PW-5) index in SubModel (dPW), the *sRPE (EWMA)* (TL-2) index in SubModel (dTL), the *urine protein* (PR-1) and *urobilinogen* (PR-2) indices in SubModel (wU), and the *squat 1RM* (PP-1) index in SubModel (wP) had significantly higher weights (*p* < 0.01). In contrast, the *sleep (EWMA)* (PW-3) and *desire (EWMA)* (PW-5) indices in SubModel (dPW), the *TM (sRPE)* (TL-1) index in SubModel (dTL), the *urine ketones* (PR-6) index in SubModel (wU), and the *MVJ* (PP-4) index in SubModel (wP) had significantly lower weights (*p* < 0.01).

When there was a risk of mild LENCI, the *menses* (PW-1) and *sleep (EWMA)* (PW-3) indices in SubModel (dPW), the *urobilinogen* (PR-2) and *urine blood* (PR-5) indices in SubModel (wU), and the *squat 1RM* (PP-1) and 5.8 m *× 6 shuttle run* (PP-2) indices in SubModel (wP) had significantly higher weights (*p* < 0.01). In contrast, the weights of the *fatigue (EWMA)* (PW-2) and *MS (EWMA)* (PW-4) indices in SubModel (dPW), the *urine protein* (PR-1), *pH* (PR-3), *SG* (PR-4) and *urine ketones* (PR-6) indices in SubModel (wU), and the 15 m *× 17 shuttle run* (PP-3) and *MVJ* (PP-4) indices in SubModel (wP) were significantly decreased (*p* < 0.01).

### Network visualization of injury severities

**FIGURE 9:**
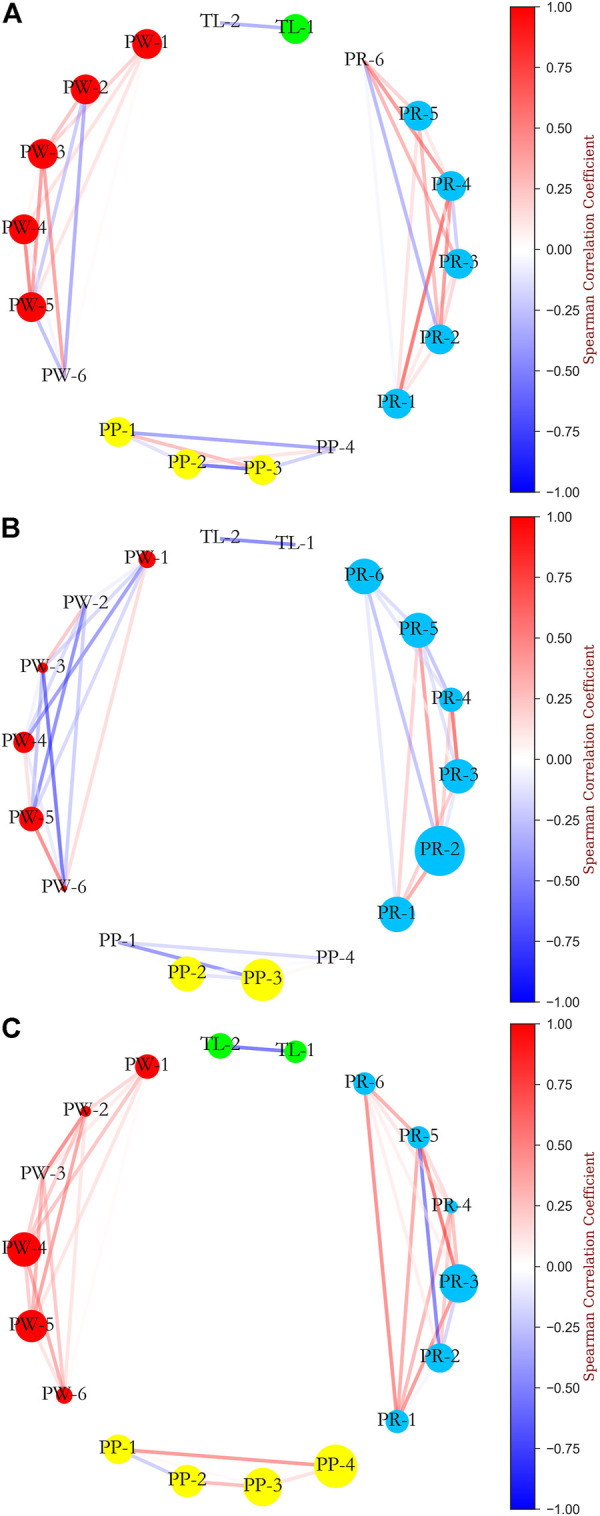
Network analysis of the relationship between SHAP values of independent variables: **(A)** non injured; **(B)** minimal LENCI risk versus non injured; **(C)** mild LENCI risk versus non injured.

To facilitate the observation of differences in the patterns of different levels of LENCI risk, we calculated the statistics of Welch’s *t*-test for each variable in the minimal and mild LENCI risk with the non-injured case, using the nodes of the non-injured network as standard nodes, and calculated the node size of each variable in the network by the Welch’s *t*-test statistics. The network was visualised to construct the network for the three cases of no impairment, minimal LENCI risk and mild LENCI (Figure 9). Larger nodes in Figures 9B,C indicate a positive Welch’s *t*-test statistic for that node compared to no LENCI risk.

We found differences in the overall means of the different variables and in the strength of the associations between the variables in each case. Among them, the overall means of *menses* (PW-1), *fatigue (EWMA)* (PW-2), *sleep (EWMA)* (PW-3), *desire (EWMA)* (PW-6), *squat 1RM* (PP-1), *MVJ* (PP -4), *TM (sRPE)* (TL-1), and *sRPE (EWMA)* (TL-2) decreased significantly in minimal LENCI risk (*p* < 0.05). The decrease in the *MS (EWMA)* (PW-4) index was marginally significant (*p* < 0.1). The overall mean values of the 15 m *× 17 shuttle run* (PP-3) and *urobilinogen* (PR-2) increased significantly (*p* < 0.05). While the overall mean values of the *fatigue (EWMA)* (PW-2), *sleep (EWMA)* (PW-3), *desire (EWMA)* (PW-6), and *SG* (PR-4) indices showed significant decreases (*p* < 0.05) in the risk of mild LENCI, the overall mean values of 15 m *× 17 shuttle run* (PP-3), *MVJ* (PP-4), and *pH* (PR-3) indices showed significant increases (*p* < 0.05). The changes in the remaining indices were not significant (*p* > 0.1).

## Discussion

This study investigates the modelling method of sports injury risk prediction models based on data from multiple sources in training practice. To a certain extent, this research work can fill the gaps in existing studies and provide the necessary reference for preventing non-contact injuries to the lower extremity of youth female basketball players in Fujian Province. There are two main findings: firstly, the study proposes a LENCI risk prediction model based on multimodal fusion and machine learning algorithms, which can effectively predict LENCI risk for different injury severities levels. Secondly, by performing feature attribution analysis and network visualisation analysis on the model, differences in LENCI risk patterns with different severity were identified.

### Advantages of multimodal modelling strategies

In recent years, the application of machine learning algorithms in sports injury risk prediction has become a hot topic of interest in sports science research. Some scholars have succeeded in exploring the effectiveness of machine learning algorithms in sports injury risk prediction by using various machine learning algorithms to model the prediction of sports injury risk (Colby et al., 2017; López-valenciano et al., 2017; Carey et al., 2018; Rossi et al., 2018; Ruddy et al., 2018). However, we noted that most of the existing research reports used data types that were too homogeneous in terms of data dimensions or single time points. For example, longitudinal observational study designs were used to obtain long-term GPS data, sRPE and other players’ data and model injury risk prediction (Carey et al., 2018; Rossi et al., 2018; Bryan et al., 2020). Alternatively, a cross-sectional study design was used to obtain pregame athletic quality assessment data and model injury prediction during the season (López-valenciano et al., 2017; Ayala et al., 2019; Jauhiainen et al., 2020; Ruiz-Pérez et al., 2021). The former captured changes in athletes’ indices prior to the onset of injury and could effectively provide a real-time assessment of injury risk daily or even per session. However, as sports injuries result from multifactorial interactions, simply focusing on changes in a single dimension does not capture a complete pattern of injury risk. The latter allowed for multidimensional data at a single point, but its drawbacks were also evident. This means that injury risk prediction models constructed from cross-sectional data only provided a staged assessment of injury risk and could not assess potential injury risk in real-time. In addition, the associations obtained through predictive models constructed using cross-sectional data could be logically flawed, i.e., the associations in the population did not reflect the associations in the individual. In view of these findings, modelling strategies for multi-source data are necessary, given that data in sports training practice are multi-source.

We constructed a LENCI risk prediction model based on previous studies using a decision-level fusion strategy and machine learning algorithms. This model was able to predict non-injured with an approximate 99.3% precision and 99.8% recall, minimal LENCI risk with an approximate 93.2% precision and 91.7% recall, and mild LENCI risk with an approximate 90.0% precision and 90.0% recall. By performing a decision curve analysis of the model, we observed that the fusion model proposed in this study leads to a higher net benefit rate for people with potential LENCI risk, which is a good indication of the practical application of the model in training practice. By comparing this modelling scheme with a traditional data integration scheme, we found that the mean values of Precision and Recall for the prediction models constructed by this modelling scheme improved by 8.2 and 20.3%, respectively, with the standard deviation of precision increasing by approximately 1.6% and the standard deviation of recall decreasing by approximately 5.0%. This showed that compared with the data integration scheme, the prediction model constructed by the multimodal fusion modelling strategy could effectively reduce the missed diagnosis rate and the misdiagnosis rate. The effectiveness of the modelling scheme in predicting the risk of LENCI at different injury severity levels was confirmed.

It should be noted that other multimodal fusion strategies still exist, such as data-level fusion and intermediate-level fusion (Atrey et al., 2010). Since the decision-level fusion strategy used in this study is to fuse the prediction results of different submodels, it can make the errors of different submodels often disconnected from and unaffected by each other without causing further accumulation of errors (Ramachandram and Taylor, 2017; Murphy, 2019). This is important for decision-making in training practice.

### Risk patterns of the lower extremity non-contact injury

The ultimate goal of sports injury risk assessment studies is not just to predict the occurrence of sports injuries but also to reduce the risk of injury by identifying potential injury risks and adjusting intervention measures promptly (Ruddy et al., 2019; De Leeuw et al., 2021). However, what conditions are athletes prone to injury? How can adjustment plans be created for specific situations? These are the two major problems facing sports injury prevention practice. This study identified differences in LENCI risk patterns for different injury severities levels by performing a feature attribution analysis and network visualisation of the model. Specifically, the weights of SubModel (dPW) and SubModel (wPP) in the dFusionModel increased significantly when athletes were at risk of Minimal LENCI compared to non-injured, while the weight of SubModel (dTL) in the FusionModel decreased significantly. This result may indicate that perceived wellness status and physical performance are potentially essential contributors to the risk of Minimal LENCI.

In contrast, when athletes are at risk of Mild LENCI, the weights of SubModel (dPW) and SubModel (wPP) in the dFusionModel decrease significantly, while the weight of SubModel (dTL) increases significantly, implying that training load may be an essential cause of Mild LENCI risk. This phenomenon is consistent with the view of (Bittencourt et al., 2016), who stated that sports injuries are the result of a combination of factors interacting in a linear or non-linear manner, leading to the same kind of injury problem, which may present different injury risk patterns depending on the specific sport, injury types, and injury severity. The view is consistent with that of physics. According to physics, this is probably since all organisms are open systems (as they exchange matter and energy with their environment without losing their identity). Open systems interact fully with their environment and constantly evolve, producing similar injury outcomes from different relationships between risk factors (Philippe and Mansi, 1998; Rickles et al., 2007; Bittencourt et al., 2016). However, previous studies have reported more focus on a specific injury factor’s relationship or direct effect on injury outcomes without focusing on specific injury risk patterns changes. Further research on injury risk patterns is still needed.

After analysing the differences in the values of each index in the risk of LENCI with different injury severities, we found that there was a tendency for the athletes’ perceived well-being indices and physical performance tests to become worse when there was a risk of minimal LENCI compared to when there was no risk of LENCI. We speculate that this might be due to a negative impact on the athletes’ sports performance and physiological status as a result of their prolonged overtraining (Hooper et al., 1995; Halson et al., 2002; Nederhof et al., 2008; Slivka et al., 2010; Laux et al., 2015; Lathlean et al., 2020). When there was a risk of mild LENCI, the athletes’ perceived well-being indices showed a trend of deterioration, consistent with previous findings. The Squat 1RM and 5.8 m × 6 Shuttle Run scores in the physical performance test remained relatively unchanged, the 15 m × 17 Shuttle Run scores deteriorated, and the MVJ scores improved. This phenomenon is inconsistent with the changes that occurred when there was a risk of minimal LENCI. The reason for this may be the mismatch between fatigue accumulation due to training load and recovery capacity, resulting in a decrease in the athlete’s resistance to fatigue, which causes an increase in short-term neuromuscular recruitment capacity with an increase in the local mechanical load on the joint (Rozzi et al., 1999; Gandevia, 2001; Meeusen et al., 2013; Azzam et al., 2015; McGuigan, 2016). Nevertheless, this study did not collect kinematic and kinetic parameters, so further research is needed.

In addition, we also analysed the changes in the weights of each index in each submodel. We found that urine protein and squat 1RM in the submodel increased, and the relative weights of the variables urine ketones and MVJ decreased when either minimal or mild LENCI risk occurred. The relative weight changes of the variables sRPE (EWMA), sleep (EWMA), and urine protein differed in the risk of LENCI for the two different injury severity levels. These results suggest that sRPE (EWMA), sleep (EWMA), and urine protein may be important indices to differentiate the risk of minimal LENCI from mild LENCI. However, due to the limited number of physiological indices involved and the current lack of reported studies on injury risk patterns for LENCI at different injury severity levels, this study is limited to describing the analysis results, and the information behind these results needs to be further research.

### Perspectives and practical applications

The multimodal LENCI risk prediction model proposed in this study can determine each athlete’s LENCI risk with a high precision and recall. This will help coaches periodic training programs and injury risk management for athletes. In addition, the model proposed in this study has good interpretability. We can observe differences in injury risk patterns between different injury severity levels through the model’s feature attribution analysis and network visualisation. This is essential for analysing the causal mechanisms of sports injuries, developing good training programs, and adopting targeted interventions to reduce the rate of sports injuries.

### Limitations

It is worth noting that there are still several limitations to this study. First, the amount of data used in the study was small. This is due to the limited number of high-level competitive athletes and the complicated obtaining of data. Second, the number of physiological response indices involved in this study was small due to the limitations of various factors, such as time, conditions, funding, and coach cooperation. Future work should expand on this by incorporating high-throughput testing techniques such as metabolomics. Third, the model was not validated for external validity. In this study, we conducted model construction by reviewing historical data and a stratified cross-validation approach. While this approach is effective in assessing the repeatability of the model development process and preventing overfitting of the model, validation of the model using external data is still lacking. Further research will be conducted in the future using the realistic scenario validation method suggested by (Rossi et al., 2022a). Lastly, this study did not focus on specific injury types, such as patellar tendinopathy. As the occurrence of sports injuries is unpredictable and injury data are complicated to obtain, we selected only the severity of LENCI as a predictor variable. Further research will be attempted in the future to incorporate specific disease types.

## Conclusion

This study proposes a risk prediction model for lower extremity non-contact injury based on multimodal fusion and machine learning algorithms. The model can effectively predicted the non-contact injury risk to lower extremities with different injury severities among adolescent female basketball players in Fujian Province. The method’s validity was confirmed through comparative analysis with the submodel and the traditional data integration scheme. However, the dataset used in this study involved a small sample size and few evaluation indices for each modality. We will expand the data dimensions in future research and conduct further research on specific injury problems. Although we believe that the model’s applicability still needs to be tested in training practice, this model offers valuable insights into future work on injury prevention due to its predictive performance and interpretability.

## Data Availability

The raw data supporting the conclusion of this article will be made available by the authors, without undue reservation.
