# Supplementary material for: A novel lower extremity non-contact injury risk prediction model based on multimodal fusion and interpretable machine learning
Source: Front Physiol. 2022 Sep 15;13:937546. doi: 10.3389/fphys.2022.937546 (PMC9520324; doi:10.3389/fphys.2022.937546)
Supplement: Supplementary file 1 [file Table1.DOCX]

# Supplementary File

Table S1. Hyperparameters of XGBoost and Random Forest in Submodels, Fusion Models

| Hyperparameter | XGBoost | RandomForest |
| --- | --- | --- |
| Boost | gbtree | - |
| Learning Rate | 0.4 | - |
| Max Depth | 200 | - |
| Num Boost Round | 2000 | - |
| Objective | multi:softmax | - |
| Base Score | 0.2 | - |
| Eval Metric | mlogloss | - |
| Scale Pos Weight | 10 | - |
| Random State | 11 | 11 |
| Silent | 0 | - |
| Num Class | 3 | - |
| Eta | 0.8 | - |
| N Estimators | - | 200 |
| Class Weight | - | balanced |

Table S2. Detailed results of the performance evaluation of the fusion and integration scheme in dataset A

| Model | Precision | | | Recall | | | F2-score | | |
| --- | --- | --- | --- | --- | --- | --- | --- | --- | --- |
|  | not injured | minimal LENCI  risk | mild LENCI  risk | not injured | minimal LENCI  risk | mild LENCI  risk | not injured | minimal LENCI  risk | mild LENCI  risk |
| DC | 0.9237 ± 0.0326 | 0.0637 ± 0.0219 | 0.0104 ± 0.0086 | 0.3365 ± 0.0415 | 0.3318 ± 0.1077 | 0.3000 ± 0.2449 | 0.3853 ± 0.0448 | 0.1796 ± 0.0599 | 0.0457 ± 0.0375 |
| LR | 0.9528 ± 0.0179 | 0.1088 ± 0.0477 | 0.0472 ± 0.0522 | 0.5688 ± 0.0740 | 0.4841 ± 0.2023 | 0.5833 ± 0.4425 | 0.6173 ± 0.0692 | 0.2826 ± 0.1181 | 0.1686 ± 0.1592 |
| SVM | 0.9573 ± 0.0182 | 0.4281 ± 0.2424 | 0.5732 ± 0.3897 | 0.9393 ± 0.0346 | 0.4083 ± 0.2424 | 0.7833 ± 0.3500 | 0.9426 ± 0.0277 | 0.3889 ± 0.2157 | 0.6635 ± 0.3266 |
| KNN | 0.9511 ± 0.0124 | 0.1938 ± 0.0761 | 0.2174 ± 0.1958 | 0.8297 ± 0.0404 | 0.3992 ± 0.1437 | 0.7833 ± 0.2693 | 0.8511 ± 0.0334 | 0.3246 ± 0.1205 | 0.4617 ± 0.2429 |
| NB | 0.9612 ± 0.0230 | 0.1303 ± 0.0520 | 0.1405 ± 0.1546 | 0.6207 ± 0.1196 | 0.6492 ± 0.2508 | 0.6500 ± 0.3905 | 0.6652 ± 0.1137 | 0.3581 ± 0.1360 | 0.3274 ± 0.2730 |
| DT | 0.9502 ± 0.0154 | 0.2786 ± 0.2213 | 0.4254 ± 0.4705 | 0.8601 ± 0.1223 | 0.3545 ± 0.1969 | 0.5000 ± 0.3873 | 0.8738 ± 0.1036 | 0.3036 ± 0.1660 | 0.3511 ± 0.3059 |
| RF | 0.9483 ± 0.0140 | 0.5152 ± 0.3708 | 0.5810 ± 0.4571 | 0.9649 ± 0.0378 | 0.2909 ± 0.1666 | 0.5500 ± 0.4153 | 0.9612 ± 0.0296 | 0.3047 ± 0.1784 | 0.5030 ± 0.3780 |
| XGBoost | 0.9483 ± 0.0131 | 0.4863 ± 0.3789 | 0.4333 ± 0.4099 | 0.9226 ± 0.0672 | 0.3265 ± 0.1697 | 0.5500 ± 0.4153 | 0.9268 ± 0.0543 | 0.3130 ± 0.1817 | 0.4708 ± 0.3706 |
| dFusionModel | 0.9932 ± 0.0205 | 0.9317 ± 0.1790 | 0.9000 ± 0.3000 | 0.9976 ± 0.0055 | 0.9167 ± 0.2500 | 0.9000 ± 0.3000 | 0.9967 ± 0.0085 | 0.9171 ± 0.2429 | 0.9000 ± 0.3000 |
